# Supplementary material for: Iterative improvement in the automatic modular design of robot swarms
Source: PeerJ Comput Sci. 2020 Dec 7;6:e322. doi: 10.7717/peerj-cs.322 (PMC7924708; doi:10.7717/peerj-cs.322)
Supplement: Supplemental Information 3 [file peerj-cs-06-322-s003.zip › argos3/doc/api/standalone/a00626_source.html]

ARGoS: plugins/simulator/physics\_engines/pointmass3d/pointmass3d\_model.h Source File


- Main Page
- Related Pages
- Namespaces
- Classes
- Files

- File List
- File Members

# plugins/simulator/physics\_engines/pointmass3d/pointmass3d\_model.h

Go to the documentation of this file.

```
00001 
00007 #ifndef POINTMASS3D_MODEL_H
00008 #define POINTMASS3D_MODEL_H
00009 
00010 namespace argos {
00011    class CPointMass3DEngine;
00012 }
00013 
00014 #include <argos3/core/simulator/physics_engine/physics_model.h>
00015 #include <argos3/plugins/simulator/physics_engines/pointmass3d/pointmass3d_engine.h>
00016 #include <argos3/core/utility/math/vector3.h>
00017 #include <argos3/core/utility/math/quaternion.h>
00018 
00019 namespace argos {
00020 
00021    class CPointMass3DModel : public CPhysicsModel {
00022 
00023    public:
00024 
00025       typedef std::map<std::string, CPointMass3DModel*> TMap;
00026 
00027    public:
00028 
00029       CPointMass3DModel(CPointMass3DEngine& c_engine,
00030                         CEmbodiedEntity& c_entity);
00031 
00032       virtual ~CPointMass3DModel() {}
00033 
00034       virtual void MoveTo(const CVector3& c_position,
00035                           const CQuaternion& c_orientation);
00036 
00037       virtual void Reset();
00038 
00039       virtual void Step() = 0;
00040       virtual void UpdateFromEntityStatus() = 0;
00041 
00042       virtual bool IsCollidingWithSomething() const;
00043 
00044       virtual bool CheckIntersectionWithRay(Real& f_t_on_ray,
00045                                             const CRay3& c_ray) const = 0;
00046 
00050       virtual void UpdateOriginAnchor(SAnchor& s_anchor);
00051 
00056       CPointMass3DEngine& GetPM3DEngine() {
00057          return m_cPM3DEngine;
00058       }
00059 
00064       const CPointMass3DEngine& GetPM3DEngine() const {
00065          return m_cPM3DEngine;
00066       }
00067 
00068    protected:
00069 
00071       CPointMass3DEngine& m_cPM3DEngine;
00072 
00074       CVector3 m_cPosition;
00075 
00077       CVector3 m_cVelocity;
00078 
00080       CVector3 m_cAcceleration;
00081 
00082    };
00083 
00084 }
00085 
00086 #endif
```

---

Generated on 10 Jul 2018 for ARGoS by 
 1.6.1
